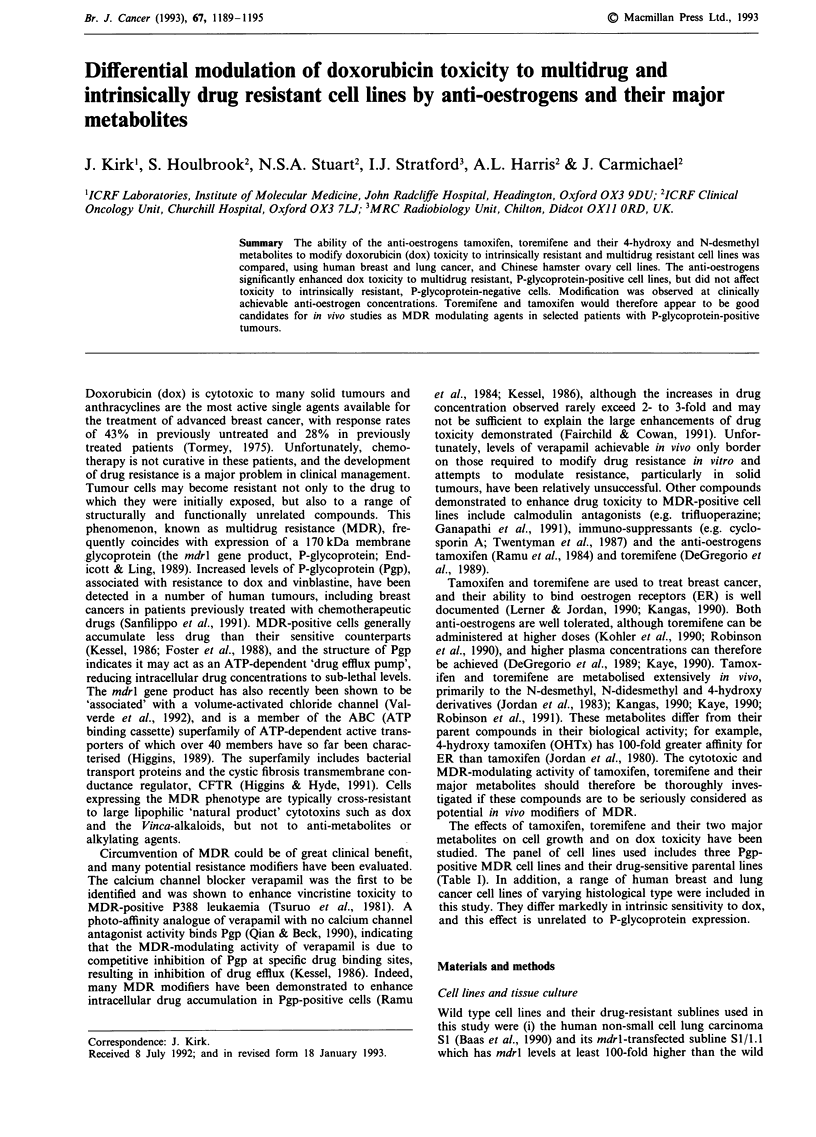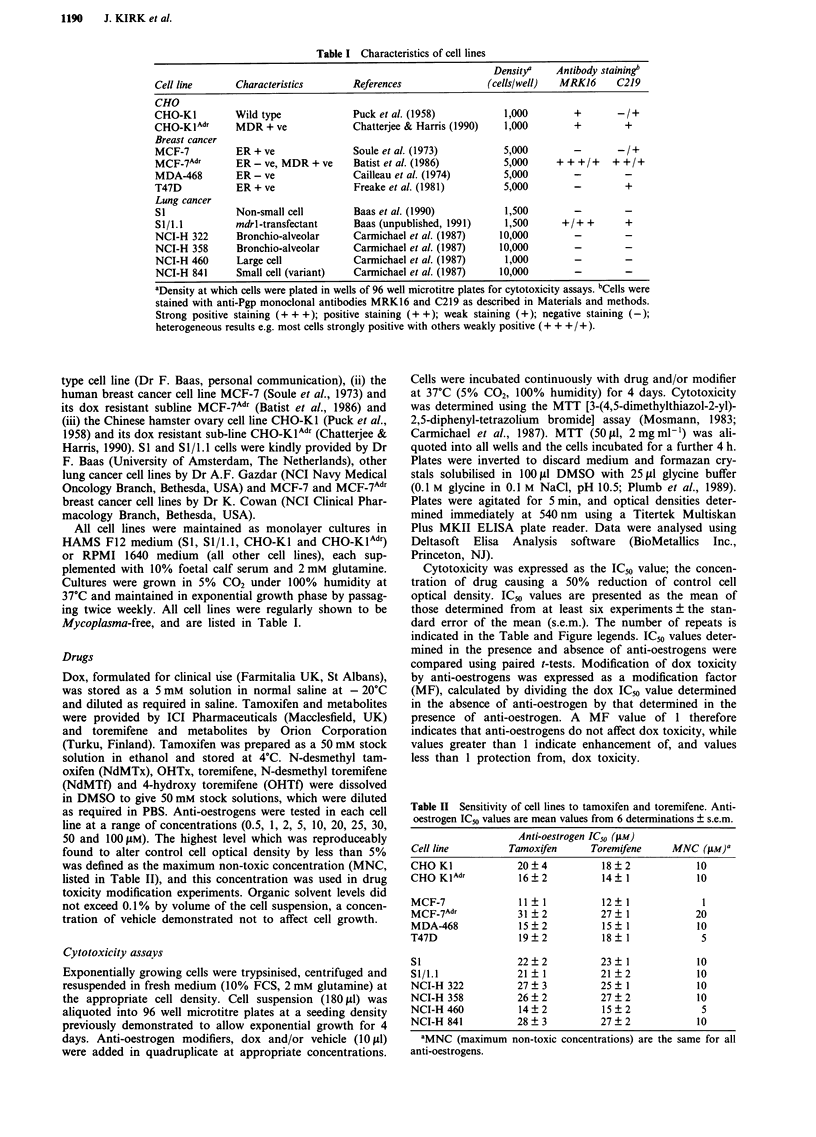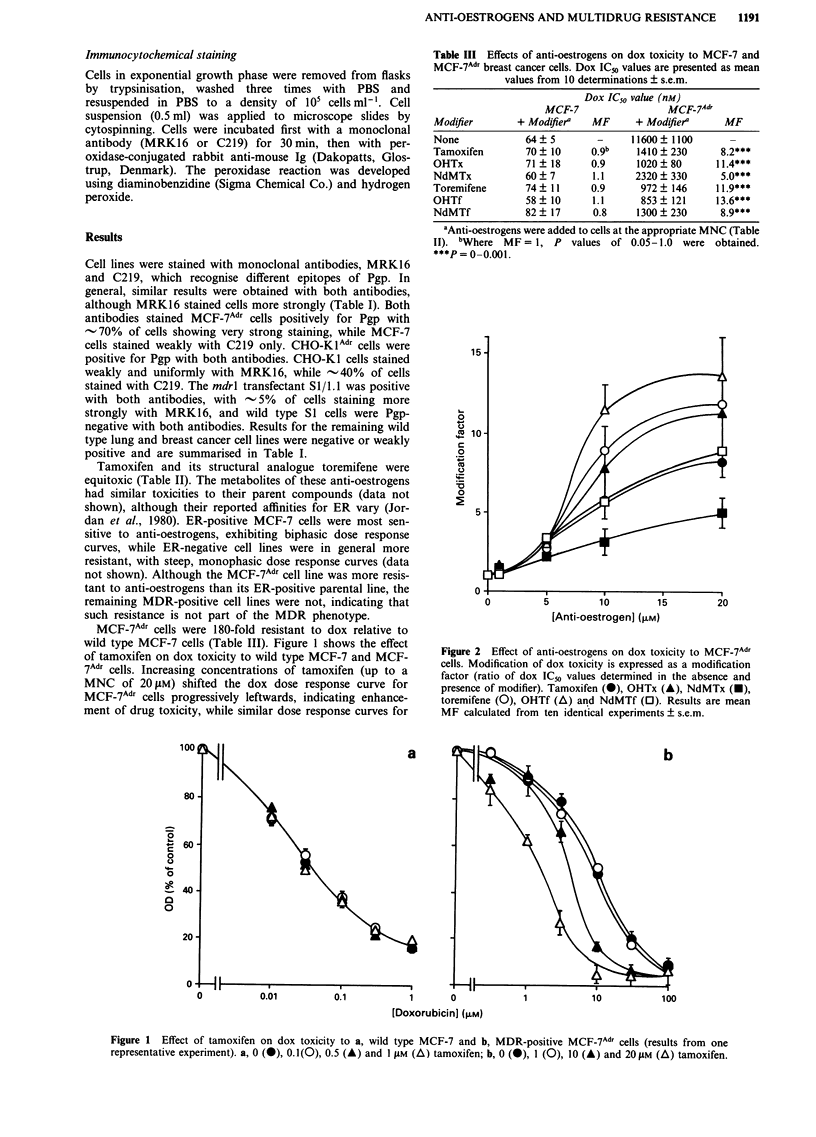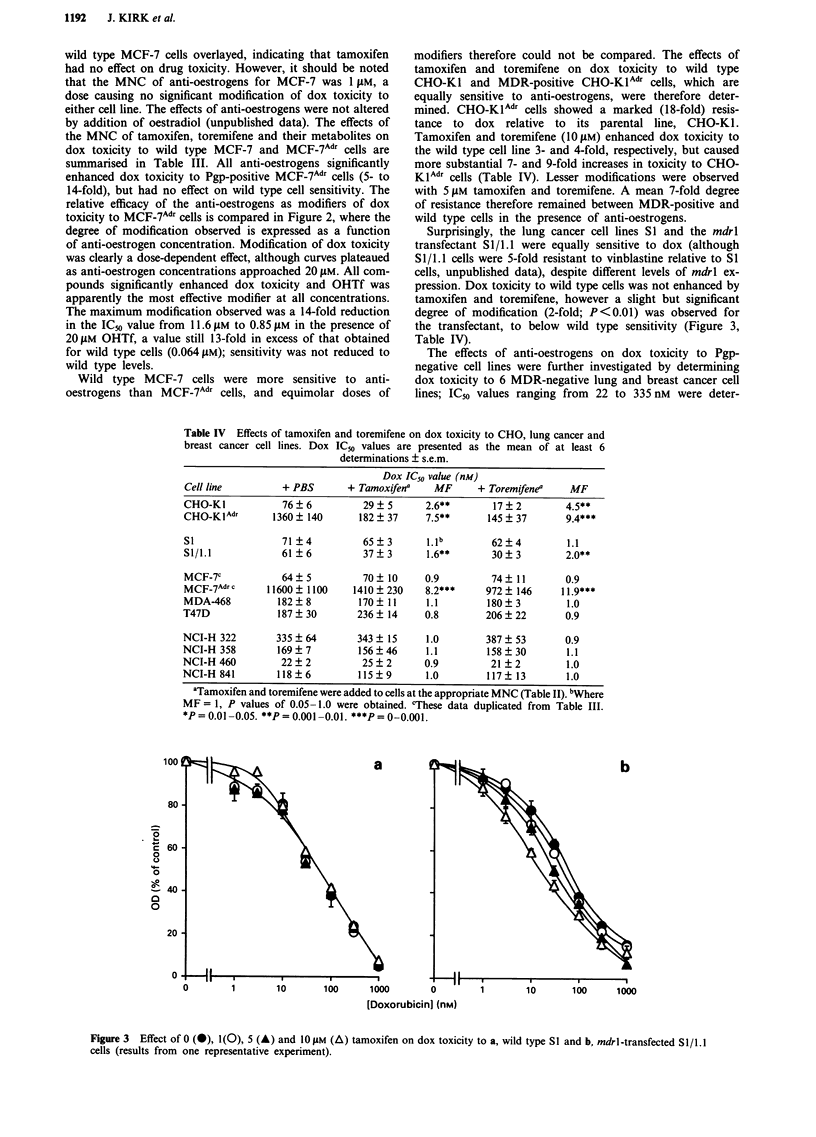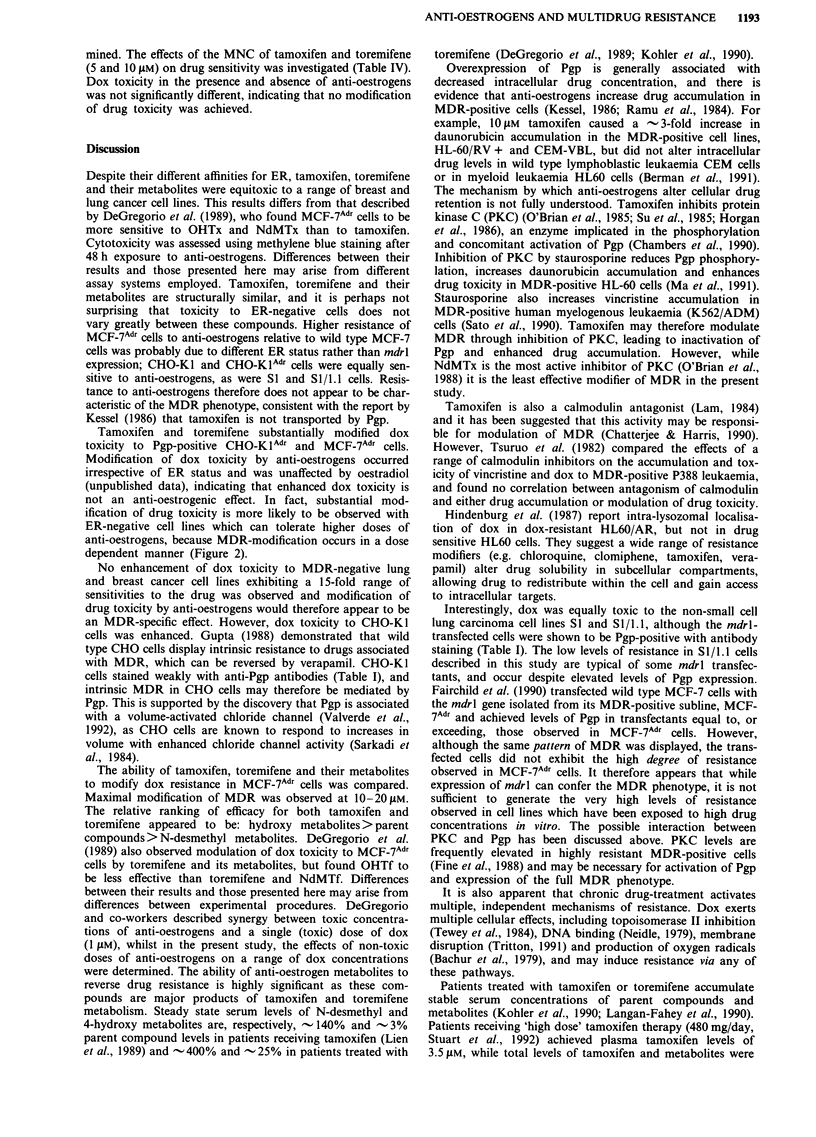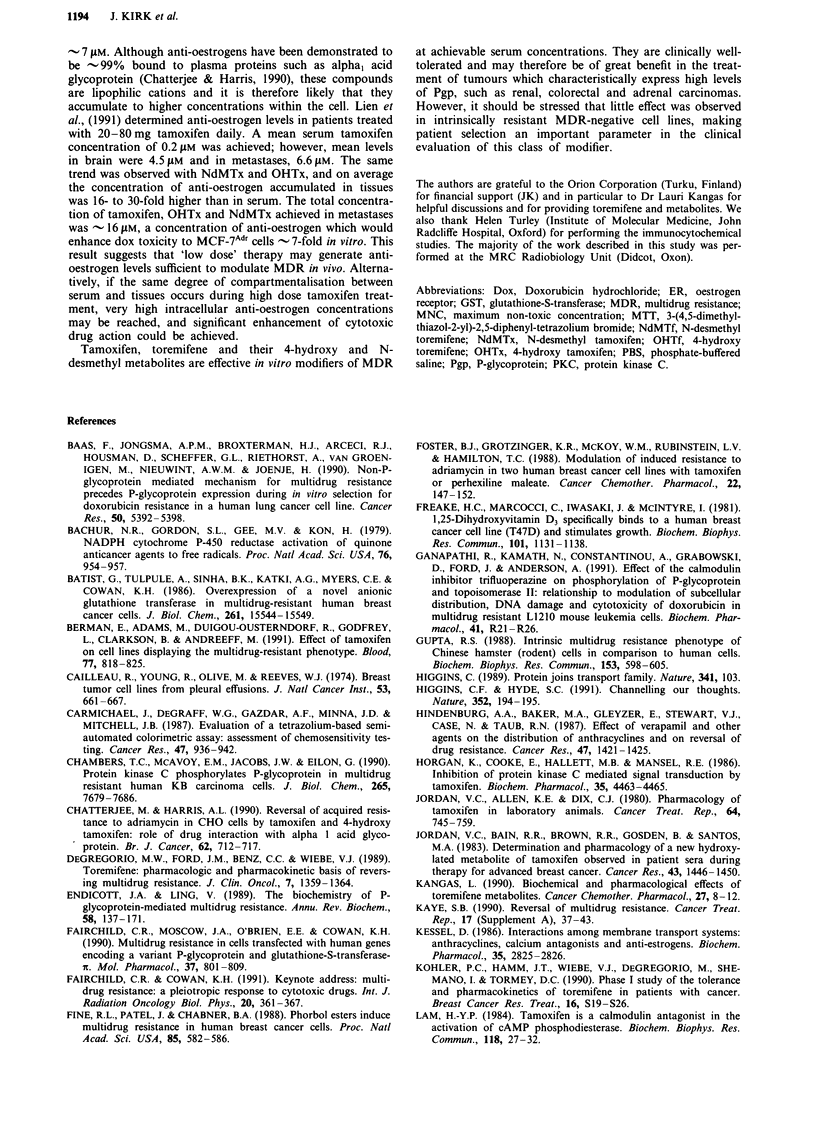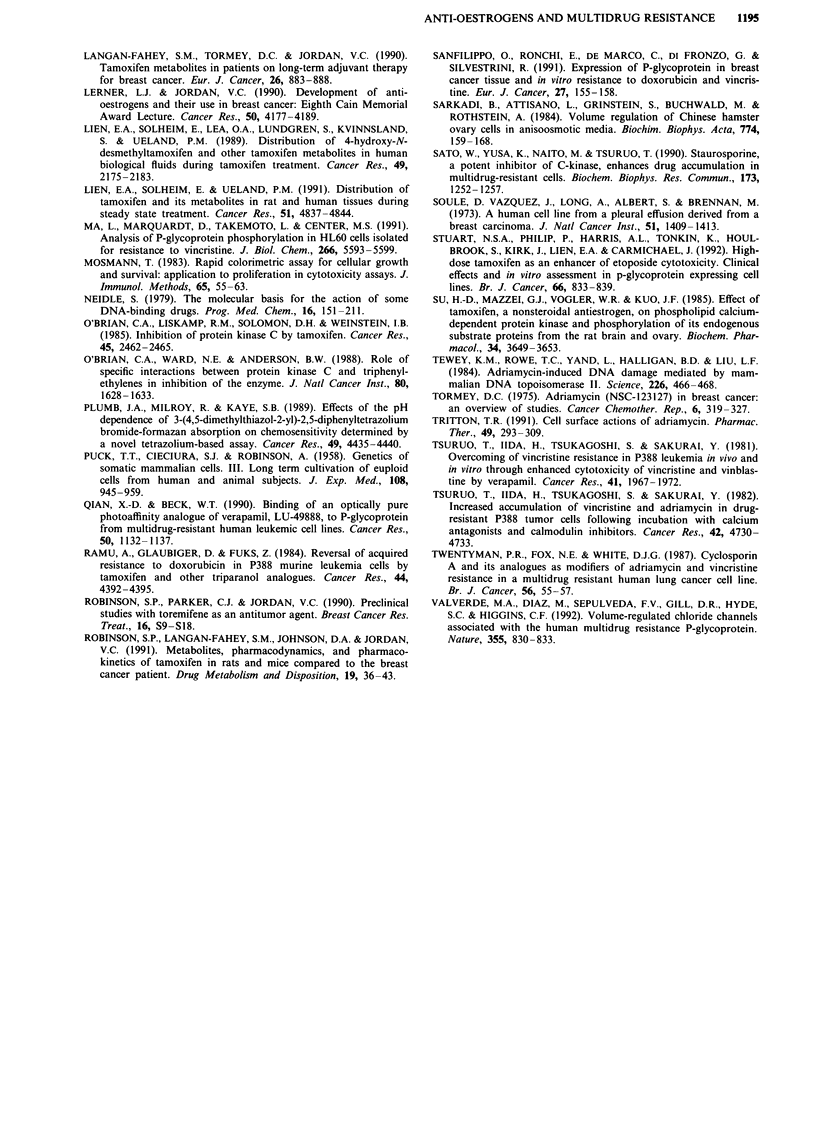# Differential modulation of doxorubicin toxicity to multidrug and intrinsically drug resistant cell lines by anti-oestrogens and their major metabolites.

**DOI:** 10.1038/bjc.1993.224

**Published:** 1993-06

**Authors:** J. Kirk, S. Houlbrook, N. S. Stuart, I. J. Stratford, A. L. Harris, J. Carmichael

**Affiliations:** ICRF Laboratory, John Radcliffe Hospital, Headington, Oxford, UK.

## Abstract

The ability of the anti-oestrogens tamoxifen, toremifene and their 4-hydroxy and N-desmethyl metabolites to modify doxorubicin (dox) toxicity to intrinsically resistant and multidrug resistant cell lines was compared, using human breast and lung cancer, and Chinese hamster ovary cell lines. The anti-oestrogens significantly enhanced dox toxicity to multidrug resistant, P-glycoprotein-positive cell lines, but did not affect toxicity to intrinsically resistant, P-glycoprotein-negative cells. Modification was observed at clinically achievable anti-oestrogen concentrations. Toremifene and tamoxifen would therefore appear to be good candidates for in vivo studies as MDR modulating agents in selected patients with P-glycoprotein-positive tumours.


Br. J. Cancer (1993), 67, 1189–1195 © Macmillan Press Ltd., 1993

### Differential modulation of doxorubicin toxicity to multidrug and intrinsically drug resistant cell lines by anti-oestrogens and their major metabolites

J. Kirk[1], S. Houlbrook[2], N.S.A. Stuart[2], I.J. Stratford[3], A.L. Harris[2] & J. Carmichael[2]

[1]*ICRF Laboratories, Institute of Molecular Medicine, John Radcliffe Hospital, Headington, Oxford OX3 9DU;* [2]*ICRF Clinical Oncology Unit, Churchill Hospital, Oxford OX3 7LJ;* [3]*MRC Radiobiology Unit, Chilton, Didcot OX11 0RD, UK.*

**Summary** The ability of the anti-oestrogens tamoxifen, toremifene and their 4-hydroxy and N-desmethyl metabolites to modify doxorubicin (dox) toxicity to intrinsically resistant and multidrug resistant cell lines was compared, using human breast and lung cancer, and Chinese hamster ovary cell lines. The anti-oestrogens significantly enhanced dox toxicity to multidrug resistant, P-glycoprotein-positive cell lines, but did not affect toxicity to intrinsically resistant, P-glycoprotein-negative cells. Modification was observed at clinically achievable anti-oestrogen concentrations. Toremifene and tamoxifen would therefore appear to be good candidates for *in vivo* studies as MDR modulating agents in selected patients with P-glycoprotein-positive tumours.

Doxorubicin (dox) is cytotoxic to many solid tumours and anthracyclines are the most active single agents available for the treatment of advanced breast cancer, with response rates of 43% in previously untreated and 28% in previously treated patients (Tormey, 1975). Unfortunately, chemotherapy is not curative in these patients, and the development of drug resistance is a major problem in clinical management. Tumour cells may become resistant not only to the drug to which they were initially exposed, but also to a range of structurally and functionally unrelated compounds. This phenomenon, known as multidrug resistance (MDR), frequently coincides with expression of a 170 kDa membrane glycoprotein (the *mdr*1 gene product, P-glycoprotein; Endicott & Ling, 1989). Increased levels of P-glycoprotein (Pgp), associated with resistance to dox and vinblastine, have been detected in a number of human tumours, including breast cancers in patients previously treated with chemotherapeutic drugs (Sanfilippo *et al.*, 1991). MDR-positive cells generally accumulate less drug than their sensitive counterparts (Kessel, 1986; Foster *et al.*, 1988), and the structure of Pgp indicates it may act as an ATP-dependent 'drug efflux pump', reducing intracellular drug concentrations to sub-lethal levels. The *mdr*1 gene product has also recently been shown to be 'associated' with a volume-activated chloride channel (Valverde *et al.*, 1992), and is a member of the ABC (ATP binding cassette) superfamily of ATP-dependent active transporters of which over 40 members have so far been characterised (Higgins, 1989). The superfamily includes bacterial transport proteins and the cystic fibrosis transmembrane conductance regulator, CFTR (Higgins & Hyde, 1991). Cells expressing the MDR phenotype are typically cross-resistant to large lipophilic 'natural product' cytotoxins such as dox and the *Vinca*-alkaloids, but not to anti-metabolites or alkylating agents.

Circumvention of MDR could be of great clinical benefit, and many potential resistance modifiers have been evaluated. The calcium channel blocker verapamil was the first to be identified and was shown to enhance vincristine toxicity to MDR-positive P388 leukaemia (Tsuruo *et al.*, 1981). A photo-affinity analogue of verapamil with no calcium channel antagonist activity binds Pgp (Qian & Beck, 1990), indicating that the MDR-modulating activity of verapamil is due to competitive inhibition of Pgp at specific drug binding sites, resulting in inhibition of drug efflux (Kessel, 1986). Indeed, many MDR modifiers have been demonstrated to enhance intracellular drug accumulation in Pgp-positive cells (Ramu *et al.*, 1984; Kessel, 1986), although the increases in drug concentration observed rarely exceed 2- to 3-fold and may not be sufficient to explain the large enhancements of drug toxicity demonstrated (Fairchild & Cowan, 1991). Unfortunately, levels of verapamil achievable *in vivo* only border on those required to modify drug resistance *in vitro* and attempts to modulate resistance, particularly in solid tumours, have been relatively unsuccessful. Other compounds demonstrated to enhance drug toxicity to MDR-positive cell lines include calmodulin antagonists (e.g. trifluoperazine; Ganapathi *et al.*, 1991), immuno-suppressants (e.g. cyclosporin A; Twentyman *et al.*, 1987) and the anti-oestrogens tamoxifen (Ramu *et al.*, 1984) and toremifene (DeGregorio *et al.*, 1989).

Tamoxifen and toremifene are used to treat breast cancer, and their ability to bind oestrogen receptors (ER) is well documented (Lerner & Jordan, 1990; Kangas, 1990). Both anti-oestrogens are well tolerated, although toremifene can be administered at higher doses (Kohler *et al.*, 1990; Robinson *et al.*, 1990), and higher plasma concentrations can therefore be achieved (DeGregorio *et al.*, 1989; Kaye, 1990). Tamoxifen and toremifene are metabolised extensively *in vivo*, primarily to the N-desmethyl, N-didesmethyl and 4-hydroxy derivatives (Jordan *et al.*, 1983); Kangas, 1990; Kaye, 1990; Robinson *et al.*, 1991). These metabolites differ from their parent compounds in their biological activity; for example, 4-hydroxy tamoxifen (OHTx) has 100-fold greater affinity for ER than tamoxifen (Jordan *et al.*, 1980). The cytotoxic and MDR-modulating activity of tamoxifen, toremifene and their major metabolites should therefore be thoroughly investigated if these compounds are to be seriously considered as potential *in vivo* modifiers of MDR.

The effects of tamoxifen, toremifene and their two major metabolites on cell growth and on dox toxicity have been studied. The panel of cell lines used includes three Pgp-positive MDR cell lines and their drug-sensitive parental lines (Table I). In addition, a range of human breast and lung cancer cell lines of varying histological type were included in this study. They differ markedly in intrinsic sensitivity to dox, and this effect is unrelated to P-glycoprotein expression.

### Materials and methods

### Cell lines and tissue culture

Wild type cell lines and their drug-resistant sublines used in this study were (i) the human non-small cell lung carcinoma S1 (Baas *et al.*, 1990) and its *mdr*1-transfected subline S1/1.1 which has *mdr*1 levels at least 100-fold higher than the wild

Correspondence: J. Kirk.
Received 8 July 1992; and in revised form 18 January 1993.

**Table I** Characteristics of cell lines

| Cell line | Characteristics | References | Density[a] (cells/well) | Antibody staining[b] MRK16 | C219 |
|---|---|---|---|---|---|
| *CHO* | | | | | |
| CHO-K1 | Wild type | Puck *et al.* (1958) | 1,000 | + | –/+ |
| CHO-K1$^{Adr}$ | MDR + ve | Chatterjee & Harris (1990) | 1,000 | + | + |
| *Breast cancer* | | | | | |
| MCF-7 | ER + ve | Soule *et al.* (1973) | 5,000 | – | –/+ |
| MCF-7$^{Adr}$ | ER – ve, MDR + ve | Batist *et al.* (1986) | 5,000 | + + +/+ | + +/+ |
| MDA-468 | ER – ve | Cailleau *et al.* (1974) | 5,000 | – | – |
| T47D | ER + ve | Freake *et al.* (1981) | 5,000 | – | + |
| *Lung cancer* | | | | | |
| S1 | Non-small cell | Baas *et al.* (1990) | 1,500 | – | – |
| S1/1.1 | *mdr*1-transfectant | Baas (unpublished, 1991) | 1,500 | +/+ + | + |
| NCI-H 322 | Bronchio-alveolar | Carmichael *et al.* (1987) | 10,000 | – | – |
| NCI-H 358 | Bronchio-alveolar | Carmichael *et al.* (1987) | 10,000 | – | – |
| NCI-H 460 | Large cell | Carmichael *et al.* (1987) | 1,000 | – | – |
| NCI-H 841 | Small cell (variant) | Carmichael *et al.* (1987) | 10,000 | – | – |

[a]Density at which cells were plated in wells of 96 well microtitre plates for cytotoxicity assays. [b]Cells were stained with anti-Pgp monoclonal antibodies MRK16 and C219 as described in Materials and methods. Strong positive staining (+ + +); positive staining (+ +); weak staining (+); negative staining (–); heterogeneous results e.g. most cells strongly positive with others weakly positive (+ + +/+).

type cell line (Dr F. Baas, personal communication), (ii) the human breast cancer cell line MCF-7 (Soule *et al.*, 1973) and its dox resistant subline MCF-7$^{Adr}$ (Batist *et al.*, 1986) and (iii) the Chinese hamster ovary cell line CHO-K1 (Puck *et al.*, 1958) and its dox resistant sub-line CHO-K1$^{Adr}$ (Chatterjee & Harris, 1990). S1 and S1/1.1 cells were kindly provided by Dr F. Baas (University of Amsterdam, The Netherlands), other lung cancer cell lines by Dr A.F. Gazdar (NCI Navy Medical Oncology Branch, Bethesda, USA) and MCF-7 and MCF-7$^{Adr}$ breast cancer cell lines by Dr K. Cowan (NCI Clinical Pharmacology Branch, Bethesda, USA).

All cell lines were maintained as monolayer cultures in HAMS F12 medium (S1, S1/1.1, CHO-K1 and CHO-K1$^{Adr}$) or RPMI 1640 medium (all other cell lines), each supplemented with 10% foetal calf serum and 2 mM glutamine. Cultures were grown in 5% $CO_2$ under 100% humidity at 37°C and maintained in exponential growth phase by passaging twice weekly. All cell lines were regularly shown to be *Mycoplasma*-free, and are listed in Table I.

### *Drugs*

Dox, formulated for clinical use (Farmitalia UK, St Albans), was stored as a 5 mM solution in normal saline at – 20°C and diluted as required in saline. Tamoxifen and metabolites were provided by ICI Pharmaceuticals (Macclesfield, UK) and toremifene and metabolites by Orion Corporation (Turku, Finland). Tamoxifen was prepared as a 50 mM stock solution in ethanol and stored at 4°C. N-desmethyl tamoxifen (NdMTx), OHTx, toremifene, N-desmethyl toremifene (NdMTf) and 4-hydroxy toremifene (OHTf) were dissolved in DMSO to give 50 mM stock solutions, which were diluted as required in PBS. Anti-oestrogens were tested in each cell line at a range of concentrations (0.5, 1, 2, 5, 10, 20, 25, 30, 50 and 100 μM). The highest level which was reproduceably found to alter control cell optical density by less than 5% was defined as the maximum non-toxic concentration (MNC, listed in Table II), and this concentration was used in drug toxicity modification experiments. Organic solvent levels did not exceed 0.1% by volume of the cell suspension, a concentration of vehicle demonstrated not to affect cell growth.

### *Cytotoxicity assays*

Exponentially growing cells were trypsinised, centrifuged and resuspended in fresh medium (10% FCS, 2 mM glutamine) at the appropriate cell density. Cell suspension (180 μl) was aliquoted into 96 well microtitre plates at a seeding density previously demonstrated to allow exponential growth for 4 days. Anti-oestrogen modifiers, dox and/or vehicle (10 μl) were added in quadruplicate at appropriate concentrations. Cells were incubated continuously with drug and/or modifier at 37°C (5% $CO_2$, 100% humidity) for 4 days. Cytotoxicity was determined using the MTT [3-(4,5-dimethylthiazol-2-yl)-2,5-diphenyl-tetrazolium bromide] assay (Mosmann, 1983; Carmichael *et al.*, 1987). MTT (50 μl, 2 mg ml$^{-1}$) was aliquoted into all wells and the cells incubated for a further 4 h. Plates were inverted to discard medium and formazan crystals solubilised in 100 μl DMSO with 25 μl glycine buffer (0.1 M glycine in 0.1 M NaCl, pH 10.5; Plumb *et al.*, 1989). Plates were agitated for 5 min, and optical densities determined immediately at 540 nm using a Titertek Multiskan Plus MKII ELISA plate reader. Data were analysed using Deltasoft Elisa Analysis software (BioMetallics Inc., Princeton, NJ).

Cytotoxicity was expressed as the $IC_{50}$ value; the concentration of drug causing a 50% reduction of control cell optical density. $IC_{50}$ values are presented as the mean of those determined from at least six experiments ± the standard error of the mean (s.e.m.). The number of repeats is indicated in the Table and Figure legends. $IC_{50}$ values determined in the presence and absence of anti-oestrogens were compared using paired *t*-tests. Modification of dox toxicity by anti-oestrogens was expressed as a modification factor (MF), calculated by dividing the dox $IC_{50}$ value determined in the absence of anti-oestrogen by that determined in the presence of anti-oestrogen. A MF value of 1 therefore indicates that anti-oestrogens do not affect dox toxicity, while values greater than 1 indicate enhancement of, and values less than 1 protection from, dox toxicity.

**Table II** Sensitivity of cell lines to tamoxifen and toremifene. Anti-oestrogen $IC_{50}$ values are mean values from 6 determinations ± s.e.m.

| Cell line | Anti-oestrogen $IC_{50}$ (μM) Tamoxifen | Toremifene | MNC (μM)[a] |
|---|---|---|---|
| CHO K1 | 20 ± 4 | 18 ± 2 | 10 |
| CHO K1$^{Adr}$ | 16 ± 2 | 14 ± 1 | 10 |
| MCF-7 | 11 ± 1 | 12 ± 1 | 1 |
| MCF-7$^{Adr}$ | 31 ± 2 | 27 ± 1 | 20 |
| MDA-468 | 15 ± 2 | 15 ± 1 | 10 |
| T47D | 19 ± 2 | 18 ± 1 | 5 |
| S1 | 22 ± 2 | 23 ± 1 | 10 |
| S1/1.1 | 21 ± 1 | 21 ± 2 | 10 |
| NCI-H 322 | 27 ± 3 | 25 ± 1 | 10 |
| NCI-H 358 | 26 ± 2 | 27 ± 2 | 10 |
| NCI-H 460 | 14 ± 2 | 15 ± 2 | 5 |
| NCI-H 841 | 28 ± 3 | 27 ± 2 | 10 |

[a]MNC (maximum non-toxic concentrations) are the same for all anti-oestrogens.

*Immunocytochemical staining*

Cells in exponential growth phase were removed from flasks by trypsinisation, washed three times with PBS and resuspended in PBS to a density of $10^5$ cells $ml^{-1}$. Cell suspension (0.5 ml) was applied to microscope slides by cytospinning. Cells were incubated first with a monoclonal antibody (MRK16 or C219) for 30 min, then with peroxidase-conjugated rabbit anti-mouse Ig (Dakopatts, Glostrup, Denmark). The peroxidase reaction was developed using diaminobenzidine (Sigma Chemical Co.) and hydrogen peroxide.

### Results

Cell lines were stained with monoclonal antibodies, MRK16 and C219, which recognise different epitopes of Pgp. In general, similar results were obtained with both antibodies, although MRK16 stained cells more strongly (Table I). Both antibodies stained MCF-$7^{Adr}$ cells positively for Pgp with ~70% of cells showing very strong staining, while MCF-7 cells stained weakly with C219 only. CHO-K$1^{Adr}$ cells were positive for Pgp with both antibodies. CHO-K1 cells stained weakly and uniformly with MRK16, while ~40% of cells stained with C219. The *mdr*1 transfectant S1/1.1 was positive with both antibodies, with ~5% of cells staining more strongly with MRK16, and wild type S1 cells were Pgp-negative with both antibodies. Results for the remaining wild type lung and breast cancer cell lines were negative or weakly positive and are summarised in Table I.

Tamoxifen and its structural analogue toremifene were equitoxic (Table II). The metabolites of these anti-oestrogens had similar toxicities to their parent compounds (data not shown), although their reported affinities for ER vary (Jordan *et al.*, 1980). ER-positive MCF-7 cells were most sensitive to anti-oestrogens, exhibiting biphasic dose response curves, while ER-negative cell lines were in general more resistant, with steep, monophasic dose response curves (data not shown). Although the MCF-$7^{Adr}$ cell line was more resistant to anti-oestrogens than its ER-positive parental line, the remaining MDR-positive cell lines were not, indicating that such resistance is not part of the MDR phenotype.

MCF-$7^{Adr}$ cells were 180-fold resistant to dox relative to wild type MCF-7 cells (Table III). Figure 1 shows the effect of tamoxifen on dox toxicity to wild type MCF-7 and MCF-$7^{Adr}$ cells. Increasing concentrations of tamoxifen (up to a MNC of 20 μM) shifted the dox dose response curve for MCF-$7^{Adr}$ cells progressively leftwards, indicating enhancement of drug toxicity, while similar dose response curves for

**Table III** Effects of anti-oestrogens on dox toxicity to MCF-7 and MCF-$7^{Adr}$ breast cancer cells. Dox $IC_{50}$ values are presented as mean values from 10 determinations ± s.e.m.

| | Dox $IC_{50}$ value (nM) | | | |
|---|---|---|---|---|
| *Modifier* | *MCF-7 + Modifier*[a] | *MF* | *MCF-7*$^{Adr}$ *+ Modifier*[a] | *MF* |
| None | 64 ± 5 | – | 11600 ± 1100 | – |
| Tamoxifen | 70 ± 10 | 0.9[b] | 1410 ± 230 | 8.2*** |
| OHTx | 71 ± 18 | 0.9 | 1020 ± 80 | 11.4*** |
| NdMTx | 60 ± 7 | 1.1 | 2320 ± 330 | 5.0*** |
| Toremifene | 74 ± 11 | 0.9 | 972 ± 146 | 11.9*** |
| OHTf | 58 ± 10 | 1.1 | 853 ± 121 | 13.6*** |
| NdMTf | 82 ± 17 | 0.8 | 1300 ± 230 | 8.9*** |

[a]Anti-oestrogens were added to cells at the appropriate MNC (Table II). [b]Where MF = 1, *P* values of 0.05–1.0 were obtained. ****P* = 0–0.001.

**Figure 2** Effect of anti-oestrogens on dox toxicity to MCF-$7^{Adr}$ cells. Modification of dox toxicity is expressed as a modification factor (ratio of dox $IC_{50}$ values determined in the absence and presence of modifier). Tamoxifen (●), OHTx (▲), NdMTx (■), toremifene (○), OHTf (△) and NdMTf (□). Results are mean MF calculated from ten identical experiments ± s.e.m.

**Figure 1** Effect of tamoxifen on dox toxicity to **a**, wild type MCF-7 and **b**, MDR-positive MCF-$7^{Adr}$ cells (results from one representative experiment). **a**, 0 (●), 0.1(○), 0.5 (▲) and 1 μM (△) tamoxifen; **b**, 0 (●), 1 (○), 10 (▲) and 20 μM (△) tamoxifen.

wild type MCF-7 cells overlayed, indicating that tamoxifen had no effect on drug toxicity. However, it should be noted that the MNC of anti-oestrogens for MCF-7 was 1 μM, a dose causing no significant modification of dox toxicity to either cell line. The effects of anti-oestrogens were not altered by addition of oestradiol (unpublished data). The effects of the MNC of tamoxifen, toremifene and their metabolites on dox toxicity to wild type MCF-7 and MCF-7$^{Adr}$ cells are summarised in Table III. All anti-oestrogens significantly enhanced dox toxicity to Pgp-positive MCF-7$^{Adr}$ cells (5- to 14-fold), but had no effect on wild type cell sensitivity. The relative efficacy of the anti-oestrogens as modifiers of dox toxicity to MCF-7$^{Adr}$ cells is compared in Figure 2, where the degree of modification observed is expressed as a function of anti-oestrogen concentration. Modification of dox toxicity was clearly a dose-dependent effect, although curves plateaued as anti-oestrogen concentrations approached 20 μM. All compounds significantly enhanced dox toxicity and OHTf was apparently the most effective modifier at all concentrations. The maximum modification observed was a 14-fold reduction in the $IC_{50}$ value from 11.6 μM to 0.85 μM in the presence of 20 μM OHTf, a value still 13-fold in excess of that obtained for wild type cells (0.064 μM); sensitivity was not reduced to wild type levels.

Wild type MCF-7 cells were more sensitive to anti-oestrogens than MCF-7$^{Adr}$ cells, and equimolar doses of modifiers therefore could not be compared. The effects of tamoxifen and toremifene on dox toxicity to wild type CHO-K1 and MDR-positive CHO-K1$^{Adr}$ cells, which are equally sensitive to anti-oestrogens, were therefore determined. CHO-K1$^{Adr}$ cells showed a marked (18-fold) resistance to dox relative to its parental line, CHO-K1. Tamoxifen and toremifene (10 μM) enhanced dox toxicity to the wild type cell line 3- and 4-fold, respectively, but caused more substantial 7- and 9-fold increases in toxicity to CHO-K1$^{Adr}$ cells (Table IV). Lesser modifications were observed with 5 μM tamoxifen and toremifene. A mean 7-fold degree of resistance therefore remained between MDR-positive and wild type cells in the presence of anti-oestrogens.

Surprisingly, the lung cancer cell lines S1 and the *mdr1* transfectant S1/1.1 were equally sensitive to dox (although S1/1.1 cells were 5-fold resistant to vinblastine relative to S1 cells, unpublished data), despite different levels of *mdr1* expression. Dox toxicity to wild type cells was not enhanced by tamoxifen and toremifene, however a slight but significant degree of modification (2-fold; $P<0.01$) was observed for the transfectant, to below wild type sensitivity (Figure 3, Table IV).

The effects of anti-oestrogens on dox toxicity to Pgp-negative cell lines were further investigated by determining dox toxicity to 6 MDR-negative lung and breast cancer cell lines; $IC_{50}$ values ranging from 22 to 335 nM were deter-

**Table IV** Effects of tamoxifen and toremifene on dox toxicity to CHO, lung cancer and breast cancer cell lines. Dox $IC_{50}$ values are presented as the mean of at least 6 determinations ± s.e.m.

| | Dox $IC_{50}$ value (nM) | | | | |
|---|---|---|---|---|---|
| *Cell line* | *+ PBS* | *+ Tamoxifen*[a] | *MF* | *+ Toremifene*[a] | *MF* |
| CHO-K1 | 76 ± 6 | 29 ± 5 | 2.6** | 17 ± 2 | 4.5** |
| CHO-K1$^{Adr}$ | 1360 ± 140 | 182 ± 37 | 7.5** | 145 ± 37 | 9.4*** |
| S1 | 71 ± 4 | 65 ± 3 | 1.1[b] | 62 ± 4 | 1.1 |
| S1/1.1 | 61 ± 6 | 37 ± 3 | 1.6** | 30 ± 3 | 2.0** |
| MCF-7[c] | 64 ± 5 | 70 ± 10 | 0.9 | 74 ± 11 | 0.9 |
| MCF-7$^{Adr}$[c] | 11600 ± 1100 | 1410 ± 230 | 8.2*** | 972 ± 146 | 11.9*** |
| MDA-468 | 182 ± 8 | 170 ± 11 | 1.1 | 180 ± 3 | 1.0 |
| T47D | 187 ± 30 | 236 ± 14 | 0.8 | 206 ± 22 | 0.9 |
| NCI-H 322 | 335 ± 64 | 343 ± 15 | 1.0 | 387 ± 53 | 0.9 |
| NCI-H 358 | 169 ± 7 | 156 ± 46 | 1.1 | 158 ± 30 | 1.1 |
| NCI-H 460 | 22 ± 2 | 25 ± 2 | 0.9 | 21 ± 2 | 1.0 |
| NCI-H 841 | 118 ± 6 | 115 ± 9 | 1.0 | 117 ± 13 | 1.0 |

[a]Tamoxifen and toremifene were added to cells at the appropriate MNC (Table II). [b]Where MF = 1, *P* values of 0.05–1.0 were obtained. [c]These data duplicated from Table III. **P* = 0.01–0.05. ***P* = 0.001–0.01. ****P* = 0–0.001.

**Figure 3** Effect of 0 (●), 1(○), 5 (▲) and 10 μM (△) tamoxifen on dox toxicity to **a**, wild type S1 and **b**, *mdr1*-transfected S1/1.1 cells (results from one representative experiment).

mined. The effects of the MNC of tamoxifen and toremifene (5 and 10 μM) on drug sensitivity was investigated (Table IV). Dox toxicity in the presence and absence of anti-oestrogens was not significantly different, indicating that no modification of drug toxicity was achieved.

### Discussion

Despite their different affinities for ER, tamoxifen, toremifene and their metabolites were equitoxic to a range of breast and lung cancer cell lines. This results differs from that described by DeGregorio *et al.* (1989), who found MCF-$7^{Adr}$ cells to be more sensitive to OHTx and NdMTx than to tamoxifen. Cytotoxicity was assessed using methylene blue staining after 48 h exposure to anti-oestrogens. Differences between their results and those presented here may arise from different assay systems employed. Tamoxifen, toremifene and their metabolites are structurally similar, and it is perhaps not surprising that toxicity to ER-negative cells does not vary greatly between these compounds. Higher resistance of MCF-$7^{Adr}$ cells to anti-oestrogens relative to wild type MCF-7 cells was probably due to different ER status rather than *mdr*1 expression; CHO-K1 and CHO-$K1^{Adr}$ cells were equally sensitive to anti-oestrogens, as were S1 and S1/1.1 cells. Resistance to anti-oestrogens therefore does not appear to be characteristic of the MDR phenotype, consistent with the report by Kessel (1986) that tamoxifen is not transported by Pgp.

Tamoxifen and toremifene substantially modified dox toxicity to Pgp-positive CHO-$K1^{Adr}$ and MCF-$7^{Adr}$ cells. Modification of dox toxicity by anti-oestrogens occurred irrespective of ER status and was unaffected by oestradiol (unpublished data), indicating that enhanced dox toxicity is not an anti-oestrogenic effect. In fact, substantial modification of drug toxicity is more likely to be observed with ER-negative cell lines which can tolerate higher doses of anti-oestrogens, because MDR-modification occurs in a dose dependent manner (Figure 2).

No enhancement of dox toxicity to MDR-negative lung and breast cancer cell lines exhibiting a 15-fold range of sensitivities to the drug was observed and modification of drug toxicity by anti-oestrogens would therefore appear to be an MDR-specific effect. However, dox toxicity to CHO-K1 cells was enhanced. Gupta (1988) demonstrated that wild type CHO cells display intrinsic resistance to drugs associated with MDR, which can be reversed by verapamil. CHO-K1 cells stained weakly with anti-Pgp antibodies (Table I), and intrinsic MDR in CHO cells may therefore be mediated by Pgp. This is supported by the discovery that Pgp is associated with a volume-activated chloride channel (Valverde *et al.*, 1992), as CHO cells are known to respond to increases in volume with enhanced chloride channel activity (Sarkadi *et al.*, 1984).

The ability of tamoxifen, toremifene and their metabolites to modify dox resistance in MCF-$7^{Adr}$ cells was compared. Maximal modification of MDR was observed at 10–20 μM. The relative ranking of efficacy for both tamoxifen and toremifene appeared to be: hydroxy metabolites > parent compounds > N-desmethyl metabolites. DeGregorio *et al.* (1989) also observed modulation of dox toxicity to MCF-$7^{Adr}$ cells by toremifene and its metabolites, but found OHTf to be less effective than toremifene and NdMTf. Differences between their results and those presented here may arise from differences between experimental procedures. DeGregorio and co-workers described synergy between toxic concentrations of anti-oestrogens and a single (toxic) dose of dox (1 μM), whilst in the present study, the effects of non-toxic doses of anti-oestrogens on a range of dox concentrations were determined. The ability of anti-oestrogen metabolites to reverse drug resistance is highly significant as these compounds are major products of tamoxifen and toremifene metabolism. Steady state serum levels of N-desmethyl and 4-hydroxy metabolites are, respectively, ~140% and ~3% parent compound levels in patients receiving tamoxifen (Lien *et al.*, 1989) and ~400% and ~25% in patients treated with toremifene (DeGregorio *et al.*, 1989; Kohler *et al.*, 1990).

Overexpression of Pgp is generally associated with decreased intracellular drug concentration, and there is evidence that anti-oestrogens increase drug accumulation in MDR-positive cells (Kessel, 1986; Ramu *et al.*, 1984). For example, 10 μM tamoxifen caused a ~3-fold increase in daunorubicin accumulation in the MDR-positive cell lines, HL-60/RV+ and CEM-VBL, but did not alter intracellular drug levels in wild type lymphoblastic leukaemia CEM cells or in myeloid leukaemia HL60 cells (Berman *et al.*, 1991). The mechanism by which anti-oestrogens alter cellular drug retention is not fully understood. Tamoxifen inhibits protein kinase C (PKC) (O'Brian *et al.*, 1985; Su *et al.*, 1985; Horgan *et al.*, 1986), an enzyme implicated in the phosphorylation and concomitant activation of Pgp (Chambers *et al.*, 1990). Inhibition of PKC by staurosporine reduces Pgp phosphorylation, increases daunorubicin accumulation and enhances drug toxicity in MDR-positive HL-60 cells (Ma *et al.*, 1991). Staurosporine also increases vincristine accumulation in MDR-positive human myelogenous leukaemia (K562/ADM) cells (Sato *et al.*, 1990). Tamoxifen may therefore modulate MDR through inhibition of PKC, leading to inactivation of Pgp and enhanced drug accumulation. However, while NdMTx is the most active inhibitor of PKC (O'Brian *et al.*, 1988) it is the least effective modifier of MDR in the present study.

Tamoxifen is also a calmodulin antagonist (Lam, 1984) and it has been suggested that this activity may be responsible for modulation of MDR (Chatterjee & Harris, 1990). However, Tsuruo *et al.* (1982) compared the effects of a range of calmodulin inhibitors on the accumulation and toxicity of vincristine and dox to MDR-positive P388 leukaemia, and found no correlation between antagonism of calmodulin and either drug accumulation or modulation of drug toxicity.

Hindenburg *et al.* (1987) report intra-lysozomal localisation of dox in dox-resistant HL60/AR, but not in drug sensitive HL60 cells. They suggest a wide range of resistance modifiers (e.g. chloroquine, clomiphene, tamoxifen, verapamil) alter drug solubility in subcellular compartments, allowing drug to redistribute within the cell and gain access to intracellular targets.

Interestingly, dox was equally toxic to the non-small cell lung carcinoma cell lines S1 and S1/1.1, although the *mdr*1-transfected cells were shown to be Pgp-positive with antibody staining (Table I). The low levels of resistance in S1/1.1 cells described in this study are typical of some *mdr*1 transfectants, and occur despite elevated levels of Pgp expression. Fairchild *et al.* (1990) transfected wild type MCF-7 cells with the *mdr*1 gene isolated from its MDR-positive subline, MCF-$7^{Adr}$ and achieved levels of Pgp in transfectants equal to, or exceeding, those observed in MCF-$7^{Adr}$ cells. However, although the same *pattern* of MDR was displayed, the transfected cells did not exhibit the high *degree* of resistance observed in MCF-$7^{Adr}$ cells. It therefore appears that while expression of *mdr*1 can confer the MDR phenotype, it is not sufficient to generate the very high levels of resistance observed in cell lines which have been exposed to high drug concentrations *in vitro*. The possible interaction between PKC and Pgp has been discussed above. PKC levels are frequently elevated in highly resistant MDR-positive cells (Fine *et al.*, 1988) and may be necessary for activation of Pgp and expression of the full MDR phenotype.

It is also apparent that chronic drug-treatment activates multiple, independent mechanisms of resistance. Dox exerts multiple cellular effects, including topoisomerase II inhibition (Tewey *et al.*, 1984), DNA binding (Neidle, 1979), membrane disruption (Tritton, 1991) and production of oxygen radicals (Bachur *et al.*, 1979), and may induce resistance *via* any of these pathways.

Patients treated with tamoxifen or toremifene accumulate stable serum concentrations of parent compounds and metabolites (Kohler *et al.*, 1990; Langan-Fahey *et al.*, 1990). Patients receiving 'high dose' tamoxifen therapy (480 mg/day, Stuart *et al.*, 1992) achieved plasma tamoxifen levels of 3.5 μM, while total levels of tamoxifen and metabolites were

~7 μM. Although anti-oestrogens have been demonstrated to be ~99% bound to plasma proteins such as $alpha_1$ acid glycoprotein (Chatterjee & Harris, 1990), these compounds are lipophilic cations and it is therefore likely that they accumulate to higher concentrations within the cell. Lien *et al.*, (1991) determined anti-oestrogen levels in patients treated with 20–80 mg tamoxifen daily. A mean serum tamoxifen concentration of 0.2 μM was achieved; however, mean levels in brain were 4.5 μM and in metastases, 6.6 μM. The same trend was observed with NdMTx and OHTx, and on average the concentration of anti-oestrogen accumulated in tissues was 16- to 30-fold higher than in serum. The total concentration of tamoxifen, OHTx and NdMTx achieved in metastases was ~16 μM, a concentration of anti-oestrogen which would enhance dox toxicity to MCF-$7^{Adr}$ cells ~7-fold *in vitro*. This result suggests that 'low dose' therapy may generate anti-oestrogen levels sufficient to modulate MDR *in vivo*. Alternatively, if the same degree of compartmentalisation between serum and tissues occurs during high dose tamoxifen treatment, very high intracellular anti-oestrogen concentrations may be reached, and significant enhancement of cytotoxic drug action could be achieved.

Tamoxifen, toremifene and their 4-hydroxy and N-desmethyl metabolites are effective *in vitro* modifiers of MDR at achievable serum concentrations. They are clinically well-tolerated and may therefore be of great benefit in the treatment of tumours which characteristically express high levels of Pgp, such as renal, colorectal and adrenal carcinomas. However, it should be stressed that little effect was observed in intrinsically resistant MDR-negative cell lines, making patient selection an important parameter in the clinical evaluation of this class of modifier.

The authors are grateful to the Orion Corporation (Turku, Finland) for financial support (JK) and in particular to Dr Lauri Kangas for helpful discussions and for providing toremifene and metabolites. We also thank Helen Turley (Institute of Molecular Medicine, John Radcliffe Hospital, Oxford) for performing the immunocytochemical studies. The majority of the work described in this study was performed at the MRC Radiobiology Unit (Didcot, Oxon).

Abbreviations: Dox, Doxorubicin hydrochloride; ER, oestrogen receptor; GST, glutathione-S-transferase; MDR, multidrug resistance; MNC, maximum non-toxic concentration; MTT, 3-(4,5-dimethylthiazol-2-yl)-2,5-diphenyl-tetrazolium bromide; NdMTf, N-desmethyl toremifene; NdMTx, N-desmethyl tamoxifen; OHTf, 4-hydroxy toremifene; OHTx, 4-hydroxy tamoxifen; PBS, phosphate-buffered saline; Pgp, P-glycoprotein; PKC, protein kinase C.

### References

BAAS, F., JONGSMA, A.P.M., BROXTERMAN, H.J., ARCECI, R.J., HOUSMAN, D., SCHEFFER, G.L., RIETHORST, A., VAN GROENIGEN, M., NIEUWINT, A.W.M. & JOENJE, H. (1990). Non-P-glycoprotein mediated mechanism for multidrug resistance precedes P-glycoprotein expression during *in vitro* selection for doxorubicin resistance in a human lung cancer cell line. *Cancer Res.*, **50**, 5392–5398.

BACHUR, N.R., GORDON, S.L., GEE, M.V. & KON, H. (1979). NADPH cytochrome P-450 reductase activation of quinone anticancer agents to free radicals. *Proc. Natl Acad. Sci. USA*, **76**, 954–957.

BATIST, G., TULPULE, A., SINHA, B.K., KATKI, A.G., MYERS, C.E. & COWAN, K.H. (1986). Overexpression of a novel anionic glutathione transferase in multidrug-resistant human breast cancer cells. *J. Biol. Chem.*, **261**, 15544–15549.

BERMAN, E., ADAMS, M., DUIGOU-OUSTERNDORF, R., GODFREY, L., CLARKSON, B. & ANDREEFF, M. (1991). Effect of tamoxifen on cell lines displaying the multidrug-resistant phenotype. *Blood*, **77**, 818–825.

CAILLEAU, R., YOUNG, R., OLIVE, M. & REEVES, W.J. (1974). Breast tumor cell lines from pleural effusions. *J. Natl Cancer Inst.*, **53**, 661–667.

CARMICHAEL, J., DEGRAFF, W.G., GAZDAR, A.F., MINNA, J.D. & MITCHELL, J.B. (1987). Evaluation of a tetrazolium-based semi-automated colorimetric assay: assessment of chemosensitivity testing. *Cancer Res.*, **47**, 936–942.

CHAMBERS, T.C., MCAVOY, E.M., JACOBS, J.W. & EILON, G. (1990). Protein kinase C phosphorylates P-glycoprotein in multidrug resistant human KB carcinoma cells. *J. Biol. Chem.*, **265**, 7679–7686.

CHATTERJEE, M. & HARRIS, A.L. (1990). Reversal of acquired resistance to adriamycin in CHO cells by tamoxifen and 4-hydroxy tamoxifen: role of drug interaction with alpha 1 acid glycoprotein. *Br. J. Cancer*, **62**, 712–717.

DEGREGORIO, M.W., FORD, J.M., BENZ, C.C. & WIEBE, V.J. (1989). Toremifene: pharmacologic and pharmacokinetic basis of reversing multidrug resistance. *J. Clin. Oncol.*, **7**, 1359–1364.

ENDICOTT, J.A. & LING, V. (1989). The biochemistry of P-glycoprotein-mediated multidrug resistance. *Annu. Rev. Biochem.*, **58**, 137–171.

FAIRCHILD, C.R., MOSCOW, J.A., O'BRIEN, E.E. & COWAN, K.H. (1990). Multidrug resistance in cells transfected with human genes encoding a variant P-glycoprotein and glutathione-S-transferase-π. *Mol. Pharmacol.*, **37**, 801–809.

FAIRCHILD, C.R. & COWAN, K.H. (1991). Keynote address: multidrug resistance: a pleiotropic response to cytotoxic drugs. *Int. J. Radiation Oncology Biol. Phys.*, **20**, 361–367.

FINE, R.L., PATEL, J. & CHABNER, B.A. (1988). Phorbol esters induce multidrug resistance in human breast cancer cells. *Proc. Natl Acad. Sci. USA*, **85**, 582–586.

FOSTER, B.J., GROTZINGER, K.R., MCKOY, W.M., RUBINSTEIN, L.V. & HAMILTON, T.C. (1988). Modulation of induced resistance to adriamycin in two human breast cancer cell lines with tamoxifen or perhexiline maleate. *Cancer Chemother. Pharmacol.*, **22**, 147–152.

FREAKE, H.C., MARCOCCI, C., IWASAKI, J. & MCINTYRE, I. (1981). 1,25-Dihydroxyvitamin $D_3$ specifically binds to a human breast cancer cell line (T47D) and stimulates growth. *Biochem. Biophys. Res. Commun.*, **101**, 1131–1138.

GANAPATHI, R., KAMATH, N., CONSTANTINOU, A., GRABOWSKI, D., FORD, J. & ANDERSON, A. (1991). Effect of the calmodulin inhibitor trifluoperazine on phosphorylation of P-glycoprotein and topoisomerase II: relationship to modulation of subcellular distribution, DNA damage and cytotoxicity of doxorubicin in multidrug resistant L1210 mouse leukemia cells. *Biochem. Pharmacol.*, **41**, R21–R26.

GUPTA, R.S. (1988). Intrinsic multidrug resistance phenotype of Chinese hamster (rodent) cells in comparison to human cells. *Biochem. Biophys. Res. Commun.*, **153**, 598–605.

HIGGINS, C. (1989). Protein joins transport family. *Nature*, **341**, 103.

HIGGINS, C.F. & HYDE, S.C. (1991). Channelling our thoughts. *Nature*, **352**, 194–195.

HINDENBURG, A.A., BAKER, M.A., GLEYZER, E., STEWART, V.J., CASE, N. & TAUB, R.N. (1987). Effect of verapamil and other agents on the distribution of anthracyclines and on reversal of drug resistance. *Cancer Res.*, **47**, 1421–1425.

HORGAN, K., COOKE, E., HALLETT, M.B. & MANSEL, R.E. (1986). Inhibition of protein kinase C mediated signal transduction by tamoxifen. *Biochem. Pharmacol.*, **35**, 4463–4465.

JORDAN, V.C., ALLEN, K.E. & DIX, C.J. (1980). Pharmacology of tamoxifen in laboratory animals. *Cancer Treat. Rep.*, **64**, 745–759.

JORDAN, V.C., BAIN, R.R., BROWN, R.R., GOSDEN, B. & SANTOS, M.A. (1983). Determination and pharmacology of a new hydroxylated metabolite of tamoxifen observed in patient sera during therapy for advanced breast cancer. *Cancer Res.*, **43**, 1446–1450.

KANGAS, L. (1990). Biochemical and pharmacological effects of toremifene metabolites. *Cancer Chemother. Pharmacol.*, **27**, 8–12.

KAYE, S.B. (1990). Reversal of multidrug resistance. *Cancer Treat. Rep.*, **17** (Supplement A), 37–43.

KESSEL, D. (1986). Interactions among membrane transport systems: anthracyclines, calcium antagonists and anti-estrogens. *Biochem. Pharmacol.*, **35**, 2825–2826.

KOHLER, P.C., HAMM, J.T., WIEBE, V.J., DEGREGORIO, M., SHEMANO, I. & TORMEY, D.C. (1990). Phase I study of the tolerance and pharmacokinetics of toremifene in patients with cancer. *Breast Cancer Res. Treat.*, **16**, S19–S26.

LAM, H.-Y.P. (1984). Tamoxifen is a calmodulin antagonist in the activation of cAMP phosphodiesterase. *Biochem. Biophys. Res. Commun.*, **118**, 27–32.

LANGAN-FAHEY, S.M., TORMEY, D.C. & JORDAN, V.C. (1990). Tamoxifen metabolites in patients on long-term adjuvant therapy for breast cancer. *Eur. J. Cancer*, **26**, 883–888.

LERNER, L.J. & JORDAN, V.C. (1990). Development of antioestrogens and their use in breast cancer: Eighth Cain Memorial Award Lecture. *Cancer Res.*, **50**, 4177–4189.

LIEN, E.A., SOLHEIM, E., LEA, O.A., LUNDGREN, S., KVINNSLAND, S. & UELAND, P.M. (1989). Distribution of 4-hydroxy-*N*-desmethyltamoxifen and other tamoxifen metabolites in human biological fluids during tamoxifen treatment. *Cancer Res.*, **49**, 2175–2183.

LIEN, E.A., SOLHEIM, E. & UELAND, P.M. (1991). Distribution of tamoxifen and its metabolites in rat and human tissues during steady state treatment. *Cancer Res.*, **51**, 4837–4844.

MA, L., MARQUARDT, D., TAKEMOTO, L. & CENTER, M.S. (1991). Analysis of P-glycoprotein phosphorylation in HL60 cells isolated for resistance to vincristine. *J. Biol. Chem.*, **266**, 5593–5599.

MOSMANN, T. (1983). Rapid colorimetric assay for cellular growth and survival: application to proliferation in cytotoxicity assays. *J. Immunol. Methods*, **65**, 55–63.

NEIDLE, S. (1979). The molecular basis for the action of some DNA-binding drugs. *Prog. Med. Chem.*, **16**, 151–211.

O'BRIAN, C.A., LISKAMP, R.M., SOLOMON, D.H. & WEINSTEIN, I.B. (1985). Inhibition of protein kinase C by tamoxifen. *Cancer Res.*, **45**, 2462–2465.

O'BRIAN, C.A., WARD, N.E. & ANDERSON, B.W. (1988). Role of specific interactions between protein kinase C and triphenylethylenes in inhibition of the enzyme. *J. Natl Cancer Inst.*, **80**, 1628–1633.

PLUMB, J.A., MILROY, R. & KAYE, S.B. (1989). Effects of the pH dependence of 3-(4,5-dimethylthiazol-2-yl)-2,5-diphenyltetrazolium bromide-formazan absorption on chemosensitivity determined by a novel tetrazolium-based assay. *Cancer Res.*, **49**, 4435–4440.

PUCK, T.T., CIECIURA, S.J. & ROBINSON, A. (1958). Genetics of somatic mammalian cells. III. Long term cultivation of euploid cells from human and animal subjects. *J. Exp. Med.*, **108**, 945–959.

QIAN, X.-D. & BECK, W.T. (1990). Binding of an optically pure photoaffinity analogue of verapamil, LU-49888, to P-glycoprotein from multidrug-resistant human leukemic cell lines. *Cancer Res.*, **50**, 1132–1137.

RAMU, A., GLAUBIGER, D. & FUKS, Z. (1984). Reversal of acquired resistance to doxorubicin in P388 murine leukemia cells by tamoxifen and other triparanol analogues. *Cancer Res.*, **44**, 4392–4395.

ROBINSON, S.P., PARKER, C.J. & JORDAN, V.C. (1990). Preclinical studies with toremifene as an antitumor agent. *Breast Cancer Res. Treat.*, **16**, S9–S18.

ROBINSON, S.P., LANGAN-FAHEY, S.M., JOHNSON, D.A. & JORDAN, V.C. (1991). Metabolites, pharmacodynamics, and pharmacokinetics of tamoxifen in rats and mice compared to the breast cancer patient. *Drug Metabolism and Disposition*, **19**, 36–43.

SANFILIPPO, O., RONCHI, E., DE MARCO, C., DI FRONZO, G. & SILVESTRINI, R. (1991). Expression of P-glycoprotein in breast cancer tissue and *in vitro* resistance to doxorubicin and vincristine. *Eur. J. Cancer*, **27**, 155–158.

SARKADI, B., ATTISANO, L., GRINSTEIN, S., BUCHWALD, M. & ROTHSTEIN, A. (1984). Volume regulation of Chinese hamster ovary cells in anisoosmotic media. *Biochim. Biophys. Acta*, **774**, 159–168.

SATO, W., YUSA, K., NAITO, M. & TSURUO, T. (1990). Staurosporine, a potent inhibitor of C-kinase, enhances drug accumulation in multidrug-resistant cells. *Biochem. Biophys. Res. Commun.*, **173**, 1252–1257.

SOULE, D. VAZQUEZ, J., LONG, A., ALBERT, S. & BRENNAN, M. (1973). A human cell line from a pleural effusion derived from a breast carcinoma. *J. Natl Cancer Inst.*, **51**, 1409–1413.

STUART, N.S.A., PHILIP, P., HARRIS, A.L., TONKIN, K., HOULBROOK, S., KIRK, J., LIEN, E.A. & CARMICHAEL, J. (1992). High-dose tamoxifen as an enhancer of etoposide cytotoxicity. Clinical effects and *in vitro* assessment in p-glycoprotein expressing cell lines. *Br. J. Cancer*, **66**, 833–839.

SU, H.-D., MAZZEI, G.J., VOGLER, W.R. & KUO, J.F. (1985). Effect of tamoxifen, a nonsteroidal antiestrogen, on phospholipid calcium-dependent protein kinase and phosphorylation of its endogenous substrate proteins from the rat brain and ovary. *Biochem. Pharmacol.*, **34**, 3649–3653.

TEWEY, K.M., ROWE, T.C., YAND, L., HALLIGAN, B.D. & LIU, L.F. (1984). Adriamycin-induced DNA damage mediated by mammalian DNA topoisomerase II. *Science*, **226**, 466–468.

TORMEY, D.C. (1975). Adriamycin (NSC-123127) in breast cancer: an overview of studies. *Cancer Chemother. Rep.*, **6**, 319–327.

TRITTON, T.R. (1991). Cell surface actions of adriamycin. *Pharmac. Ther.*, **49**, 293–309.

TSURUO, T., IIDA, H., TSUKAGOSHI, S. & SAKURAI, Y. (1981). Overcoming of vincristine resistance in P388 leukemia *in vivo* and *in vitro* through enhanced cytotoxicity of vincristine and vinblastine by verapamil. *Cancer Res.*, **41**, 1967–1972.

TSURUO, T., IIDA, H., TSUKAGOSHI, S. & SAKURAI, Y. (1982). Increased accumulation of vincristine and adriamycin in drug-resistant P388 tumor cells following incubation with calcium antagonists and calmodulin inhibitors. *Cancer Res.*, **42**, 4730–4733.

TWENTYMAN, P.R., FOX, N.E. & WHITE, D.J.G. (1987). Cyclosporin A and its analogues as modifiers of adriamycin and vincristine resistance in a multidrug resistant human lung cancer cell line. *Br. J. Cancer*, **56**, 55–57.

VALVERDE, M.A., DIAZ, M., SEPULVEDA, F.V., GILL, D.R., HYDE, S.C. & HIGGINS, C.F. (1992). Volume-regulated chloride channels associated with the human multidrug resistance P-glycoprotein. *Nature*, **355**, 830–833.